# The Influence of Pathological Extracellular Matrix on the Biological Properties of Stem Cells: Possible Hints for Cell Transplantation Therapies in Spinal Cord Injury

**DOI:** 10.3390/ijms26093969

**Published:** 2025-04-23

**Authors:** Giuseppe Alastra, Corinne Quadalti, Vito Antonio Baldassarro, Alessandro Giuliani, Luciana Giardino, Laura Calzà

**Affiliations:** 1 Department of Veterinary Medical Sciences (DIMEVET), University of Bologna, Ozzano dell’Emilia, 40064 Bologna, Italy; giuseppe.alastra2@unibo.it (G.A.); vito.baldassarro2@unibo.it (V.A.B.); a.giuliani@unibo.it (A.G.); luciana.giardino@unibo.it (L.G.); 2 Department of Pharmacy and Biotechnology (FABIT), University of Bologna, 40126 Bologna, Italy; corinne.quadalti2@unibo.it; 3 Interdepartmental Centre for Industrial Research in Health Sciences and Technology ICIR HST, University of Bologna, 40126 Bologna, Italy; 4 Montecatone Rehabilitation Institute, Montecatone, 40026 Bologna, Italy

**Keywords:** extracellular matrix, spinal cord injury, rat embryonic stem cells, rat neural stem cells, 2D in vitro model, 3D in vitro model

## Abstract

Traumatic spinal cord injury (SCI) initiates a cascade of events, including persistent inflammation, which contributes to secondary injury. At a molecular level, the lesion is characterized by an altered microenvironment with changes in extracellular matrix (ECM) composition and organization, identified as a potential obstacle for effective stem cell-based cell therapies. We investigated the interactions between decellularized intact and injured rat spinal cords and rat embryonic (RESCs) and neural stem cells (NSCs) at 2 and 47 days post-lesion (dpl). Decellularized ECM was used to generate 2D coating and 3D gel in vitro platforms for cell seeding. Results showed that the 2dpl 2D coating exerted a significant negative effect on the viability of both cell types, while the 47dpl 2D coating maintained RESC pluripotency. NSCs cultured on the 2dpl 2D coating for seven days showed a severe impairment in cell growth, while maintaining a cluster formation potential and differentiation marker expression comparable to normal ECM for astrocytic and oligodendroglial lineages. Notably, when NSCs are grown in 47dpl 3D gel, the lineage turns dramatically toward an astroglial lineage. These results clearly show the detrimental effects of the SCI ECM microenvironment on stem cells, advancing the understanding of potential timings suitable for effective SCI cell-based therapies.

## 1. Introduction

Spinal cord injury (SCI) is a devastating, life-changing condition, often associated with severe functional motor and sensory impairment, and drastically reducing quality of life. The estimated worldwide prevalence of SCI is 20.6 million total cases and 250,000 to 500,000 new patients each year [1], including lesions of traumatic and nontraumatic etiology. Traumatic SCI is caused by a primary injury consequent to a sudden traumatic impact, and is characterized by a cascade of cellular and molecular events collectively defined as a “secondary injury”, consisting of ischemia, excess glutamate release and excitotoxicity, and inflammation which exacerbates tissue damage over time [2]. SCI can be clinically divided into acute (<48 h), sub-acute (<2 weeks), intermediate (<6 months), and chronic (>6 months) phases. Inflammation is particularly intense during the acute phase, but continues through the sub-acute and chronic phases, driving scar formation [2] and potentially persisting for the rest of the patient’s lifespan [3]. Inflammation and glutamate-derived excitotoxicity are the main factors responsible for progressive demyelination, which in turn leads to long-distance Wallerian degeneration [4]. The combination of these pathological events determines the clinical outcome.

The lesion microenvironment, characterized by a phenomenon known as the “microenvironment imbalance” [5], has been described as hostile to the repair attempts occurring in the central nervous system (CNS) following the lesion. A major role in this context is played by changes in the composition and topographic organization of the extracellular matrix (ECM), which consists of proteins, proteoglycans (PGs), and glycosaminoglycans (GAGs), and fills the space between neurons and glial cells, accounting for 10–20% of the total CNS volume [6]. Extracellular matrix components are secreted by both neuronal and glial cells and are spatially arranged in a tridimensional network, which is tissue-specific for biochemical composition and mechanical properties such as stiffness and topography [7].

Compared to other body districts, the CNS ECM contains a predominance of hyaluronan and chondroitin sulfate proteoglycans (CSPGs), and is organized in different domains, such as the perineural net, basal membrane, and interstitial matrix. Proteolytic enzymes, including matrix metalloproteinases (MMPs) and soluble factors (growth factors), also form part of the ECM molecular microenvironment. ECM is the key player in tissue structural integrity, but also acts as a transducer of cellular communications, thus influencing essential processes such as cell proliferation, migration, and differentiation [8,9].

In the late 1980s, the discovery of Nogo and CSPGs in the injured nervous system allowed researchers to link SCI-specific ECM composition and poor repair capability [10]. Other ECM components associated with astrocyte and scar formation, such as tenascins, semaphorins, and myelin-associated inhibitors, have subsequently been identified as hostile to axonal regrowth [11]. The increasing awareness of the role of ECM in SCI pathophysiology is also impacting cell therapy strategies, particularly with regard to the design of combined cell-biomaterial scaffolds and the timing of cell transplantation. In this context, our laboratory described changes in the expression level of 100 genes encoding for ECM proteins at 1, 8, and 45 days post-SCI in rats, evaluated in the spinal cord tissue at lesion level, and rostrally and caudally to the lesion [12]. Changes in synaptic plasticity-related genes in the same scenario were also described [13], suggesting that the survival and homing of transplanted cells may be compromised by the hostile ECM at specific timepoints following SCI.

To further explore the impact of pathological SCI ECM on the efficacy of cell therapies during the course of primary and secondary lesions, in this study, we investigated the interaction between stem cell types proposed for SCI cell therapy and ECM derived from the injured spinal cord. More in detail, we took decellularized ECM from the spinal cord of lesioned rats and from respective unlesioned controls at two timepoints (2dpl and 47dpl) to reflect the acute and chronic phases of SCI and established in vitro platforms to investigate several biological properties (adhesion, survival, differentiation, and maturation) of rat embryonic and neural stem cells in the presence of SCI-derived ECM.

## 2. Results

### 2.1. Spinal Cord Decellularization

**Figure 1:**
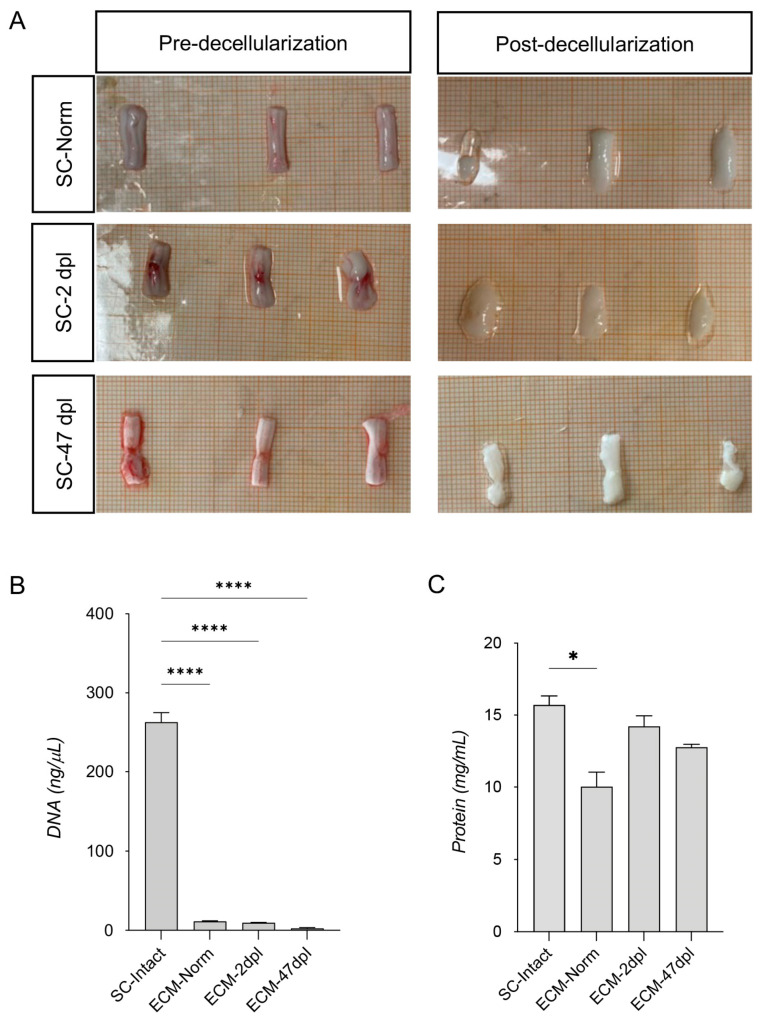
Decellularization quality controls. (**A**) Representative image of 1.5 cm core sections of SC pre- and post-decellularization protocol of all three experimental groups (SC-Normal, SC-2dpl, and SC-47dpl). (**B**,**C**) DNA (**B**), and protein (**C**) quantification to ensure correct decellularization, comparing non-decellularized (SC-intact) with decellularized tissue (SC-Normal, SC-2dpl, and SC-47dpl). Statistical analysis: one-way ANOVA followed by Dunnett’s post hoc test. Asterisks represent differences between non-decellularized tissue (SC-intact) and decellularized tissue (SC-Normal, SC-2dpl, and SC-47dpl) (* *p* < 0.05; **** *p* < 0.0001).

Decellularized SC tissue from all groups (unlesioned, 2dpl, and 47dpl) appeared translucent and compact, retaining its original shape (Figure 1A). Cell depletion was confirmed by histological staining with Hoechst, toluidine blue, and H/E staining (Supplementary Figure S1A, B, and C, respectively). The quantification of residual DNA as a decellularization quality control showed a striking depletion in decellularized compared to non-decellularized groups (one-way ANOVA, F(3,4) = 448.0, *p* < 0.0001; Dunnett’s post hoc *p* < 0.0001 for all comparisons) (Figure 1B). Total protein content was quantified by Lowry protein assay, showing a slight but still significant reduction in the comparison between intact non-decellularized SC and its decellularized counterpart only (one-way ANOVA, F(3,4) = 11.88, *p* = 0.0184; Dunnett’s post hoc *p* = 0.011) (Figure 1C). We assume this was due to the unavoidable technical variability associated with the protocol. No differences were observed among decellularized groups.

The preliminary adhesion test was performed using NSCs and ECM-Norm (no SCI) to identify the most suitable quantity of protein for the 2D coating. Results indicated that a protein quantity of 80 µg was comparable to the standard culture condition using PO-laminin coating (Supplementary Figure S2A–C); therefore, all experimental procedures were carried out using 80 µg of total proteins for the 2D coating. Similarly, a preliminary gelation test was performed to establish the most suitable protein concentration for the 3D gel experiments, which resulted as being 8 mg/mL, the lowest concentration that gelled under our experimental conditions (Supplementary Figure S2D).

### 2.2. Decellularized ECM Derived from Acutely but Not Chronically Injured Spinal Cord Negatively Impacts RESC-Sc Viability

**Figure 2:**
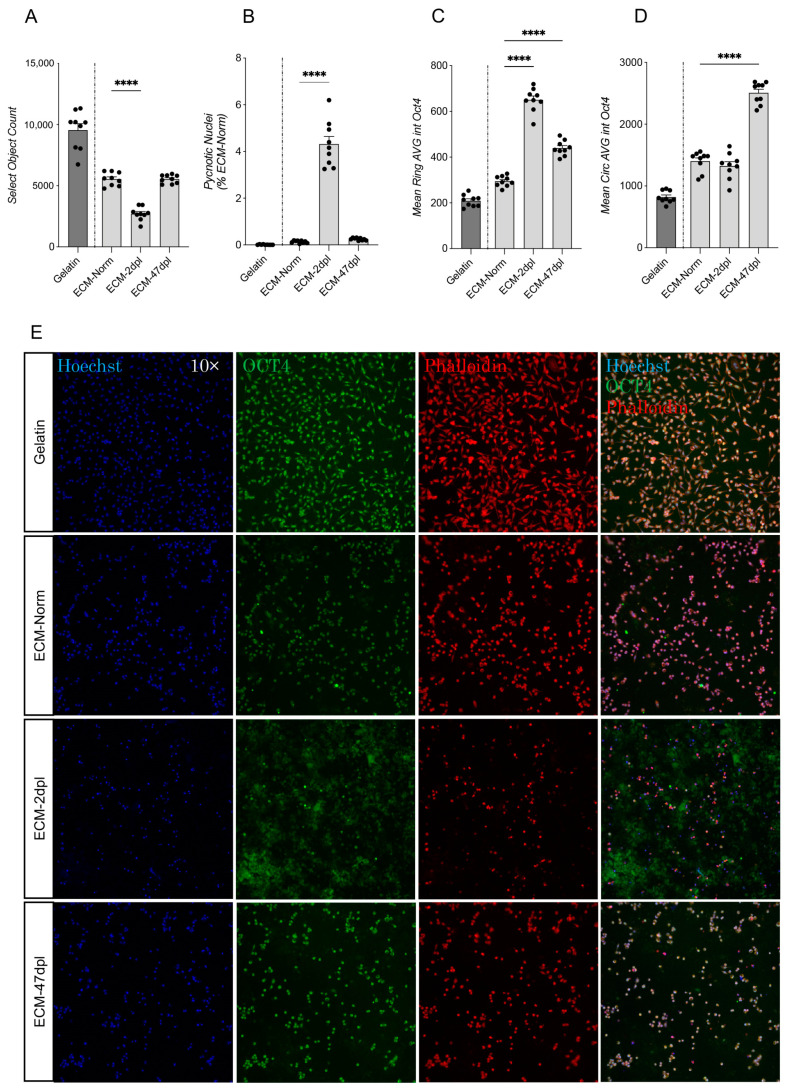
Effect of 2D ECM coating on RESC-sc at 1 DIV. (**A**–**D**) Graphs show the analysis of the number of cells per well (Select Object Count, **A**), cell death (percentage of condensed nuclei, **B**), pluripotency as positivity to OCT4 marker in perinuclear (Ring AVG, **C**) and nuclear (Circ AVG, **D**) cell compartment of RESC-sc seeded on 2D coating (gelatin, ECM-Norm, ECM-2dpl and ECM-47dpl) after 2 DIV. (**E**) Representative HCS images (magnification 10×) of RESC-sc cultures at 1 DIV, stained with Hoechst, OCT4, and Phalloidin, seeded on gelatin, ECM-Norm, ECM-2dpl, and ECM-47dpl. Statistical analysis: one-way ANOVA followed by Dunnett’s post hoc test. Asterisks represent differences between ECM-Norm (100%), ECM-2dpl, and ECM-47dpl coatings (**** *p* < 0.0001).

RESC-sc were seeded on a coating of ECM derived from the decellularized spinal cord of rats (i) with no SCI (ECM-Norm), (ii) 2 days post-lesion (ECM-2dpl, mimicking the acute phase of SCI), (iii) 47 days post-lesion (ECM-47dpl, mimicking the chronic phase), and on gelatin as the standard culture condition for RESC-sc maintenance. Data on the gelatin coating were used to confirm the status of the cells throughout the experiment and were therefore not included in the statistical analysis. Cells were grown for 24 h (1 day in vitro, 1 DIV) on the different 2D coatings and analyzed by High Content Screening (HCS). Results showed a significant impact of ECM-2dpl compared to ECM-Norm coating on both cell number (one-way ANOVA, F(2, 24) = 101.0, *p* < 0.0001; Dunnett’s post hoc, *p* < 0.0001; Figure 2A), which was reduced in ECM-2dpl, and on the percentage of pycnotic nuclei (one-way ANOVA, F(2, 24) = 157.5, *p* < 0.0001; Dunnett’s post hoc, *p* < 0.0001; Figure 2B), which was increased in ECM-2dpl, while no differences were observed in the ECM-47dpl culture condition. Interestingly, when compared to ECM-Norm, an increase in average Oct4 fluorescence intensity was observed in both the perinuclear (ring avg; one-way ANOVA, F(2, 24) = 203.5, *p* < 0.0001; Dunnett’s post hoc, *p* < 0.0001; Figure 2C) and nuclear zones (circ avg; one-way ANOVA, F(2, 24) = 116.0, *p* < 0.0001; Dunnet’s post hoc, *p* < 0.0001; Figure 2D) when cells were grown on ECM-47dpl. A significant increase in Oct4 fluorescent signal was observed in the perinuclear zone in the ECM-2dpl growth condition also (ring avg; one-way ANOVA, F(2, 24) =203.5, *p* < 0.0001; Dunnett’s post hoc, *p* < 0.0001; Figure 2C), but not in the nuclear zone. Representative, HCS-acquired images of RESC-sc when cultured in the aforementioned culture conditions for 1 DIV are shown (Figure 2E).

A significant effect of SCI ECM (2dpl and 47dpl) was observed in the adhesion potential of RESC-sc when cultured on the different experimental coatings (see Supplementary Figure S3), while comparable results in terms of viability, pycnotic nuclei, and Oct4 fluorescence intensity were obtained when RESC-sc were kept in the same experimental conditions for 48 h (2 DIV) (see Supplementary Figure S4).

### 2.3. Decellularized ECM Derived from Acutely but Not Chronically Injured Spinal Cord Negatively Impacts NSC Viability and Differentiation

**Figure 3:**
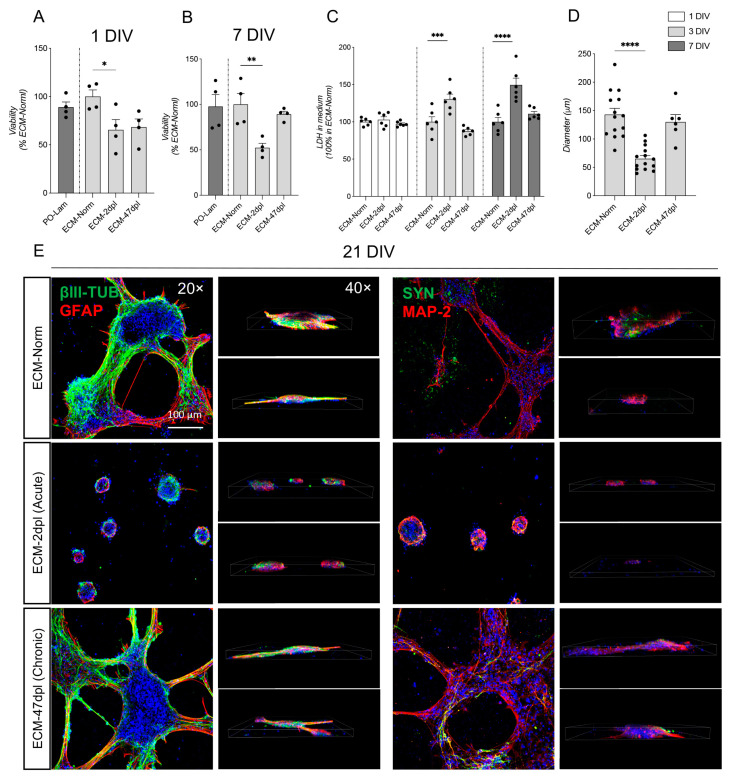
Effect of 2D ECM coating on NSCs. (**A**,**B**) Graphs show an analysis of neural stem cell (NSC) viability in a 2D coating of Poly-Ornithine/Laminin (PO-Lam), SC-Norm, SC-2dpl, and SC-47dpl at 1 and 7 DIV by MTT assay (PO-Lam is 100%), expressed as number of cells per well (**A**) and percentage of condensed nuclei (**B**). (**C**) Graph shows the analysis of LDH in culture medium of NSCs at 1, 3, and 7 DIV in a 2D coating of Dec_SC-Normal, Dec_SC-2dpl, and Dec_SC-47dpl (ECM-Norm is 100%). (**D**) Graphs show the number and diameter (μm) of clusters in NSC culture at 21 DIV, seeded on 2D coating of Dec_SC-Normal, Dec_SC-2dpl, and Dec_SC-47dpl (ECM-Norm is 100%). (**E**) Representative images of confocal z-stack as maximum intensity projection (20−, Scale bar: 100 µm), and 3D visualization (40×) of NSC clusters at 21 DIV, stained with βIII-tubulin, GFAP, MAP-2, and Synaptophysin. Statistical analysis: one-way ANOVA followed by Dunnett’s post hoc test. (**A**,**B**) Asterisks represent differences between PO-Lam (100%), Dec_SC-Norm, Dec_SC-2dpl, and Dec_SC-47dpl coatings. (**C**–**E**) Asterisks represent differences between Dec_SC-Norm coating (100%), Dec_SC-2dpl, and Dec_SC-47dpl coatings (* *p* < 0.05; ** *p* < 0.01; *** *p* < 0.001; **** *p* < 0.0001).

Neural stem cells were exposed to the same experimental conditions described above for RESC-sc, and viability at 1 and 7 DIV was evaluated (Figure 3A,B). In line with that observed with RESC-sc, NSC viability was also reduced when seeded on SCI ECM-2dpl (1 DIV: one-way ANOVA, F(3, 12) = 4.736, *p* = 0.0393; Dunnett’s post hoc, *p* = 0.0386; 7 DIV: one-way ANOVA, F(2, 9) = 10.30, *p* = 0.0047; Dunnett’s post hoc, *p* = 0.0035), but not ECM-47dpl. These results were also confirmed by LDH quantification in the culture medium (two-way ANOVA, factors: treatment F(2, 45) = 29.67, *p* < 0.0001; time F(2, 45) = 11.75, *p* < 0.0001; interaction F(4, 45) = 6.794, *p* = 0.0002; Dunnett’s post hoc, 3 DIV *p* = 0.0003; 7 DIV *p* < 0.0001) (Figure 3C).

We then examined the cell culture morphology and the presence of markers associated with neuronal (b-tub, MAP2, and synaptophysin) and astroglial (GFAP) lineages. NSCs seeded on unlesioned and 47dpl coating for 21 DIV form large, interconnected clusters containing b-tub-positive neurons and GFAP-positive astrocytes. Notably, the microtubule stabilizer MAP2 was also expressed in neurons, and the synaptic marker synaptophysin also appeared at a cell body level, as shown in z-stage confocal reconstruction. On the other hand, very small aggregates with no elongations were observed when NSCs were seeded on ECM-2dpl. This result was also quantified through cluster diameter analysis (one-way ANOVA, F(2, 31) = 21.47, *p* < 0.0001; Dunnett’s post hoc 2dpl, *p* < 0.0001) (Figure 3D,E).

**Figure 4:**
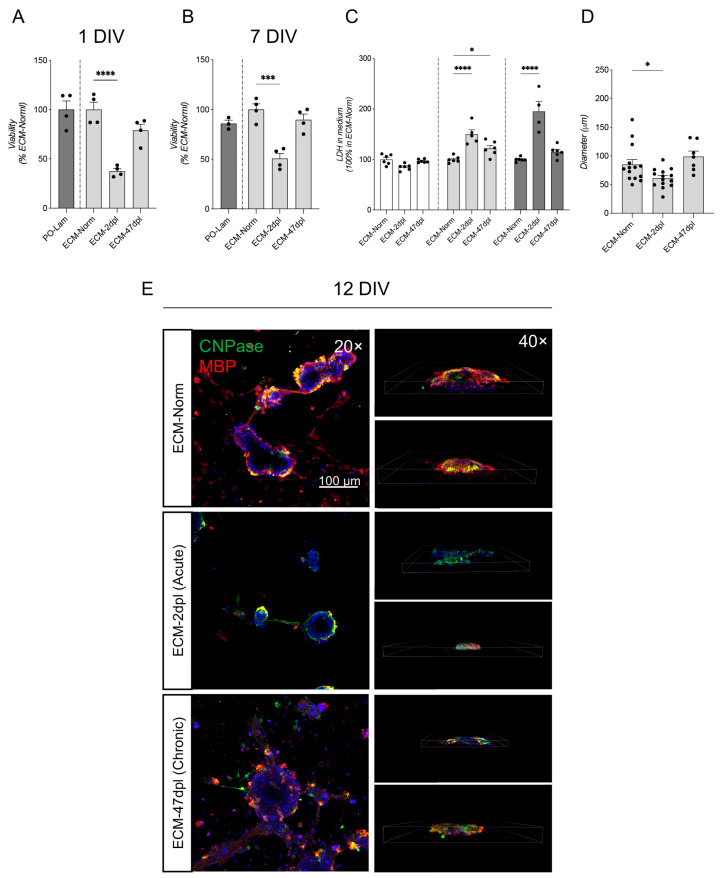
Effect of 2D ECM coating on NSC-derived OPCs. (**A**,**B**) Graphs show an analysis of the viability of OPCs in a 2D coating of Poly-Ornithine/Laminin (PO-Lam), Dec_SC-Norm, Dec_SC-2dpl, and Dec_SC-47dpl at 1 and 7 DIV by MTT assay (PO-Lam is 100%). (**C**) Graph shows an analysis of LDH in OPC culture at 1, 3, and 7 DIV in a 2D coating of Dec_SC-Normal, Dec_SC-2dpl, and Dec_SC-47dpl (ECM-Norm is 100%). (**D**) Graph shows the number and diameter (μm) of clusters in OPC culture at 21 DIV, seeded on 2D coating of Dec_SC-Normal, Dec_SC-2dpl, and Dec_SC-47dpl (ECM-Norm is 100%). (**E**) Representative images of confocal z-stack as maximum intensity projection (20×, Scale bar: 100 µm), and 3D visualization (40×) of OPC clusters at 21 DIV, stained with CNPase, MBP, and NG2. Statistical analysis: one-way ANOVA followed by Dunnett’s post hoc test. (**A**,**B**) Asterisks represent differences between PO-Lam (100%), Dec_SC-Norm, Dec_SC-2dpl and Dec_SC-47dpl coatings. (**C**,**D**) Asterisks represent differences between Dec_SC-Norm coating (100%), Dec_SC-2dpl, and Dec_SC-47dpl coatings (* *p* < 0.05; *** *p* < 0.001; **** *p* < 0.0001).

In the same experimental setup, we also investigated the NSC-derived oligodendroglial lineage and oligodendrocyte precursor cell (OPC) maturation, observing comparable results in terms of viability and differentiation as observed in the neural lineage. More in detail, cell viability was significantly impaired in cells seeded on ECM-2dpl at the investigated DIV (1 DIV: one-way ANOVA, F(2, 9) = 29.09, *p* = 0.0001; Dunnett’s post hoc, *p* < 0.0001; 7 DIV: one-way ANOVA, F(2, 9) = 22.74, *p* = 0.0003; Dunnett’s post hoc, *p* = 0.0003) (Figure 4A,B). This result was confirmed by LDH quantification (two-way ANOVA, factors: treatment F(2, 42) = 36.19, *p* < 0.0001; time F(2, 42) = 37.38 *p* < 0.0001; interaction F(4, 42) = 21.33, *p* < 0.0001; Dunnett’s post hoc, 2dpl: 3 DIV *p* < 0.0001; 7 DIV *p* < 0.0001); a slight, but significant alteration in LDH was present at 3DIV when NSCs were cultured on ECM-47dpl (two-way ANOVA, factors: treatment F(2, 42) = 36.19, *p* < 0.0001; time F(2, 42) = 37.38 *p* < 0.0001; interaction F(4, 42) = 21.33, *p* < 0.0001; Dunnett’s post hoc, 47dpl: 3 DIV *p* = 0.0264) (Figure 4C). We then investigated OPC maturation through late-differentiation markers such as CNPase and MBP. Cluster diameter was smaller in 2dpl compared to 47dpl and unlesioned ECM (one-way ANOVA, F(2, 32) = 5.812, *p* = 0.0070; Dunnett’s post hoc, *p* = 0.0376) (Figure 4D). Just as for the NSCs, OPCs seeded on unlesioned and ECM-47dpl formed interconnected clusters expressing OL differentiation markers (CNPase), and the myelin marker for mature OLs (MBP) (Figure 4E). On the other hand, OPCs seeded on ECM-2dpl expressed CNPase but not MBP, suggesting a block or delay of OL maturation.

### 2.4. Long-Term Differentiation in 3D SCI-Derived ECMs

**Figure 5:**
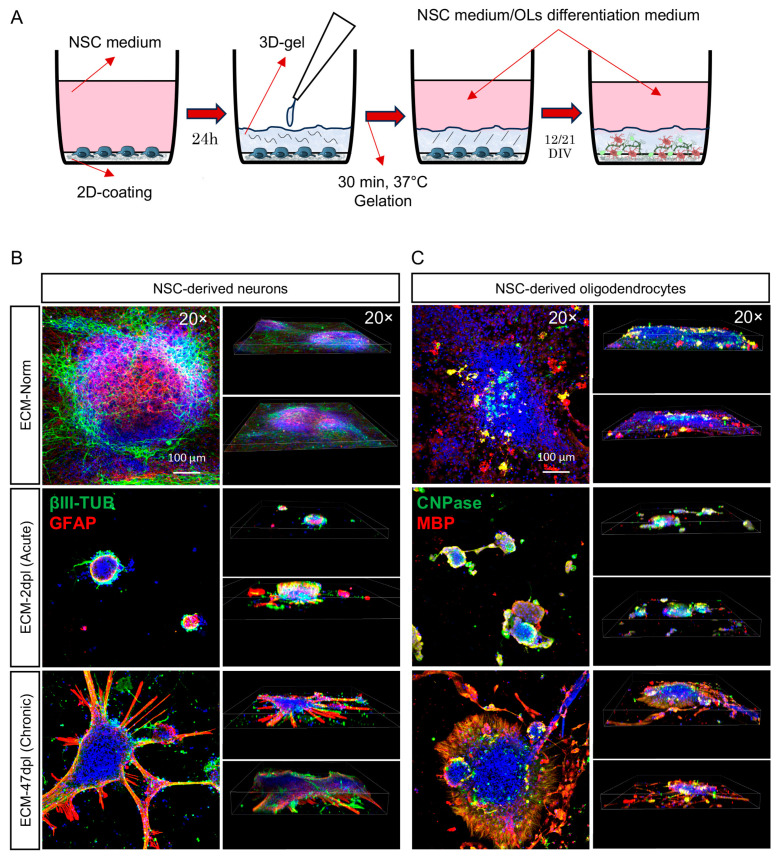
Effect of 3D ECM coating on NSCs and NSC-derived OPCs. (**A**) Schematic representation of the 3D gel experimental protocol. NSCs and OPCs were seeded on 2D ECM coating in 8-well chamber slides, the NSC medium was removed after 24 h, and 3D ECM gel was dispensed onto the cells. After dispensing, the 8-well chamber slides were incubated for 30 min at 37 °C in a humidified incubator for gelation of the 3D ECM gel. Following gelation, the NSC medium was reintroduced until the endpoint (12 or 21 DIV). (**B**,**C**) Representative images of confocal z-stack as maximum intensity projection (20×, Scale bar: 100 µm), and 3D visualization (20×) of neurospheres at 21 DIV, stained with βIII-tubulin and GFAP (**B**) and oligospheres at 12 DIV, stained with CNPase and MBP (**C**).

Given that the 2D coating systems used in the previous experiments would have been unsuited to mimicking the topographic influence of the ECM on cell differentiation, in this series of experiments, we developed a 3D gel system based on our decellularized SCI ECM. The protocol is shown in Figure 5A. Briefly, NSCs were seeded on the experimental ECM and cultured for 24 h in NSC medium, then the medium was removed and the cells were covered by the respective experimental ECM gels at 8 mg/mL. The culture was then carried out for 12 days in oligo-differentiating conditions and for 21 days in spontaneous neural differentiation conditions. NSCs in the unlesioned ECM gel developed large clusters, expressing βIII-Tub and GFAP (Figure 5B, upper panel). GFAP-positive cells were mainly grouped in the cluster core, while βIII-Tub cells extended fine and branched elongations to form a 3D net among the clusters. Cells positive for oligodendrocyte markers (CNPase and MBP) were also present in the clusters (Figure 5C upper panel). On the other hand, the ECM-2dpl gel dramatically impaired NSC growth and differentiation, as shown by the smaller and undifferentiated clusters (Figure 5B,C, central panels). NSCs in the ECM-47dpl gel were characterized by large clusters supported by a starry GFAP-positive scaffold, with few βIII-Tub-positive, poorly-branched cells observed at the periphery of this structure. Positivity for oligo-markers was also observed, forming an MBP-positive “halo” around the clusters (Figure 5B,C, bottom panels).

## 3. Discussion

Cell transplant is an advanced therapy for spinal cord injury and is regarded as a promising approach for this type of lesion. Several clinical studies based on different cell types are in progress [14]. Despite convincing preclinical results, however, the clinical relevance of cell therapies for traumatic SCI has yet to be demonstrated [14]. Several major issues are under active investigation, including the definition of cell type(s), donor characteristics [15,16], appropriate time window for cell therapy [17], and the expected biological and/or functional effects [18].

Our lab is heavily involved in preclinical studies aimed at better targeting cell therapies for SCI, the selection of cells based on donor characteristics [16], and the impact of ECM on embryonic and neural stem cell biology under physiological [19] and pathological conditions [12,20]. We have also developed original devices based on biomaterials and drugs to mitigate the hostile lesion microenvironment and promote white matter protection and repair [21,22,23].

In this study, we further extended research into the role of pathological ECM in determining stem cell fate by directly seeding stem cells on an extracellular matrix obtained from the spinal cord tissue of injured compared to uninjured rats, using a decellularization procedure [24].

Decellularization was confirmed by the almost complete absence of DNA content and histological staining, while the protein content did not differ between the decellularized samples. As described by other decellularization studies [25], the protein content after the procedure is slightly lower than in the intact spinal cord. We used rat cells for this study, specifically rat embryonic stem cells derived from blastocysts, extensively characterized by our lab [26,27,28], and neural stem cells obtained from the fetal rat brain, to differentiate between neural and oligodendroglial lineage according to protocols well established in the lab [20,29]. Both 2D and 3D strategies were included in the study, with all tools derived from rats to ensure the most faithful mimicking of ECM components and stem cell interaction, with both being from the same species.

Decellularized tissues are also emerging as a tool for regenerative medicine. This is largely due to the retention of the 3D topography and to the attitude of the cells, which are home to the pores and channels left free of endogenous cells, repopulating the scaffold [30]. Here we demonstrated that ECM derived from the spinal cord in the acute post-lesion phase (2dpl) is extremely hostile to stem cell viability and differentiation potential, in both 2D and 3D culture systems. In contrast, the viability and differentiation of stem cells when seeded on ECM obtained at the chronic phase (47dpl) is comparable to unlesioned ECM, at least in terms of the morphological features of cell cultures and differentiation markers. However, when we created a 3D system using ECM-47dpl, which more accurately reflects normal physiology, adding topographical and mechanical cues to the in vitro culture, we observed a substantial difference from the 2D system. Despite having the same chemical composition, the 3D arrangement of the matrix exerts a substantial pro-glial effect on the astrocytes and oligodendrocytes. This suggests that 3D topography and mechanical cues, such as stiffness, are powerful inducers of NSC lineage [31,32]. A thorough characterization of active molecules present in decellularized ECM, potentially at the basis of the functional differences observed between the acute (2dpl) and chronic (47dpl) phases, will be implemented. Moreover, the functional evaluation of the myelination capacity of OLs exposed to SCI-derived decellularized ECM was not performed in this first paper. As complex co-culture and readout strategies are required, this implementation will be addressed in further characterization and validation studies. In fact, the complexity of the myelination process requires suitable models to avoid the risk of oversimplifying the molecular and cellular machinery involved [33]. For example, the validation of a therapeutic strategy based on functionalized hydrogel-included MSC has been recently described in vivo, using a time-specific morphological-based approach for neurogenesis and remyelination readouts [34].

These results offer further insights into the use of cell therapies in SCI, highlighting the importance of carefully selecting the cell type and injection time window based on the expected biological effects. When validating a novel therapy for SCI, it is essential to consider the long and complex cascade of cellular and molecular events triggered by the trauma, which last for weeks and even months after the lesion, from the acute to the chronic phase, and which offer dozens of potential therapeutic targets [35]. Such events may include ischemia and electrolyte imbalances, glutamatergic excitotoxicity and free-radical production, immune cell invasion, cytokine release and inflammation-related cell death, conduction block and demyelination, and can continue for months after the lesion, with devastating functional and anatomical consequences, such as post-spinal cord injury syrinx [36]. Several cell types may contribute to controlling at least part of these events and possibly to promoting endogenous repair, as well as recruiting endogenous stem and precursor cells, but the first goal in this perspective should be to create a local microenvironment that is not hostile to the transplanted cells.

During the acute phase, the main goal is to rebalance the severe inflammation, which alters the composition and topographical structure of the ECM, as described by several authors [12,37]. However, monotherapy is insufficient for such complex conditions, as shown by the limited efficacy of pharmacological trials [38], and it is vital that novel approaches consider multiple targets, such as inflammation and remyelination [21,39]. In this regard, cells with immunomodulatory properties also exert a neuroprotective effect, as indicated by transplantation studies in the acute phase involving mesenchymal stromal cells [40], and more recently, T cells expressing reconstituted TCRs, proposed to minimize the potential adverse effects caused by the prolonged activation of self-reactive T cells [41].

When administering cell therapies based on NSCs, which theoretically exert a positive effect on endogenous repair processes (myelin repair, axonal pruning, late apoptosis limitation, etc.), careful consideration must be given to the injection time window. Our own results show that very early transplant compromises viability, while excessively late transplant risks pushing differentiation toward an astroglial lineage, thus worsening the scar formation, which is detrimental to functional recovery [42], a result observed using a differentiation protocol which included EGF and bFGF in the culture media.

An interesting result emerging from this study is that oligodendrocyte differentiation from NSCs is also favored by ECM-47dpl in the presence of bFGF and PDGF in the culture media. Notably, decellularized ECM prepared according to [24] retains its three-dimensional network structures, as indicated by the expected pores and channels of the scaffold diameters in which cells settle, thus emulating the three dimensions of the spinal cord. Mechanical cues and topography are known to play a role in oligodendrogenesis [43,44], along with chemical cues. This decellularized ECM scaffold retained fibronectin, for example, among other proteins [24]. Fibronectin inhibits outgrowth in oligodendrocytes [45], and in a previous study, we observed that the expression level of FN1, the gene encoding for fibronectin, increases in spinal cord tissue during early SCI, returning to control levels during the chronic phase [12].

These results further confirm the need to consider the extreme complexity of SCI pathophysiology in the development of new therapies, and that cell therapies would benefit from their inclusion in a polytherapy designed on the basis of the SCI phase. While further experiments are needed to clarify the molecular links between altered ECM and the viability/differentiation of stem cells, we can conclude that the acute phase of SCI is inappropriate for stem cell transplant, given the hostility of the microenvironment to both embryonic and neural stem cells. Moreover, while cell survival is maintained in the chronic phase, there is an impressive astroglial differentiation effect of the ECM, which could reflect the attitude to scar formation. However, it also strongly favors differentiation into oligodendrocytes, thus raising the issue of the astro- vs. oligo-lineage of transplanted neural stem cells, the former being negative, worsening mechanic scar formation, the latter being positive, favoring oligodendrogenesis and possibly myelin repair.

In the meantime, these results also have implications for the development of both in vitro systems for drug discovery and ATMPs for SCI. 3D bioprinting, for example, which allows the inclusion of many components, each focused on a particular target of the polytherapy (scaffolds to direct axonal growth, or the incorporation of neuroprotective molecular strategies such as neurotrophic factors, drugs, cells, or derivative products such as exosomes), will constitute a powerful tool [46], as soon as the methods meet the reproducibility standard required for drug discovery. 3D bioprinting also shows promise for the customization of implants for SCI [47], and it is crucial, in our opinion, that pathological ECM matrix be considered in these approaches.

## 4. Materials and Methods

### 4.1. Animals and Surgery

All animal protocols described herein were carried out according to European Community Council Directives 2010/63/EU, approved by the Italian Ministry of Health (Legislative Decree 26/2014, authorization no. 574/2015-PR). Animal protocols also complied with the ARRIVE guidelines and the NIH Guide for the Care and Use of Laboratory Animals. Female CD-Sprague Dawley rats (230–280 gr body weight) were used (Charles River Laboratories, Lecco, Italy).

The animals underwent a contusive spinal cord lesion at the thoracic (T9) level. Surgery was carried out according to a standardized procedure [21]. Briefly, all animals were individually housed in cages, with food and water ad libitum. On the day of surgery, rats were premedicated with enrofloxacin and tramadol (5 mg/kg, s.c.), then anesthetized with isoflurane (1–3%) in O_2_. The animals were immobilized on a spinal stereotaxic table, and a 4 cm longitudinal midline dorsal incision was made from T7 to T11. The soft tissues were dissected layer by layer to completely expose the spinous processes from T8 to T10, and the T9 spinous process and lamina were removed to expose the spinal canal and spinal dura mater. Spinal cord injury was induced using an Impact One Impactor (Leica Biosystems, Wetzlar, Germany) at T9 level using a 1.5 mm tip with a force of 1 N (0.75 m/s), 0 s support time, and 1.5 mm impact depth. The rats were treated with antibiotics and painkillers (enrofloxacin 5 mg/kg and tramadol 5 mg/kg s.c.) for 3dpl, and unlesioned animals were used as a control. Lesioned (N = 7 for each time point) and control rats (N = 3) were then sacrificed at 2dpl and 47dpl.

### 4.2. Spinal Cord Decellularization and Histology

Spinal cord decellularization was carried out according to Guo et al. [24], with some modifications. Segments measuring 1.5 cm in length were dissected from the spinal cord along the rostro-caudal axis, from 0.75 cm above to 0.75 cm below T9 level, from each experimental group (unlesioned, 2dpl, and 47dpl), and the dissected tissues were washed in cold PBS 1×. From this step onwards, all procedures were carried out under a laminar flow hood to maintain sterility. Washed tissues were placed in a shaking Petri dish (200 rpm) in Triton X-100 1%/PBS 1× for 3 h, rinsed three times in PBS 0.01% (1 h per rinse) and then in Sodium Deoxycholate 1%/PBS 1× for 12 h. At the end of incubation, tissues were rinsed three times in PBS 0.01% (1 h per rinse) and further incubated in Triton X-100 1%/PBS 1× for 3 h. Finally, tissues were washed three times with PBS 0.01% (1 h per rinse) and kept in this solution at +4 °C until use.

To analyze the quality of the decellularized product, spinal cord segments (intact and decellularized) were fixed using two different fixatives: (i) 10% formalin (16% paraformaldehyde in bdH_2_O at 55–60 °C, clarified with 2 M NaOH, 14% Picric Acid, bdH_2_O) dissolved in Sorensen Buffer 0.4 M pH 6.9 (84% Na_2_HPO_4_·2H_2_O and 16% NaH_2_PO_4_·2H_2_O) for 30 min, and (ii) 10% formalin dissolved in bdH_2_O for 90 min. Both fixatives were filtered prior to use. After fixation, tissues were washed three times in 5% sucrose (composed of a 1:1 solution of 10% sucrose dissolved in 0.2 M Sorensen Buffer pH 7.4) and macroscopically cut into cross sections, which were snap frozen in liquid nitrogen. Sections were subsequently cut coronally into 14 µm fine sections using a Leica CM1950 cryostat (Leica Biosystems, Wetzlar, Germany) and evaluated via hematoxylin/eosin and toluidine blue histologic staining, associated with Hoechst 33258 nuclear dye (1 μg/mL; Invitrogen, Waltham, MA, USA, Cat no. H3569). Following histological staining, the spinal cord slices were immersed in xylene for 5 min, and slides were mounted using Eukitt^®^ quick-hardening mounting medium (Sigma-Aldrich, St. Louis, MO, USA).

The decellularized SC was homogenized (at a ratio of mg of tissue to mL of PBS 1× of 1:10 weight/volume) using T 25 basic ULTRA-TURRAX^®^ homogenizer and lyophilized to powder using Concentrator Plus (Eppendorf^®^, Hamburg, Germany) overnight, then stored at −20 °C until use. For the 2D ECM coating and the 3D ECM gel, the powder was digested in 1 mg/mL pepsin (Sigma-Aldrich, cat no. S-P7012-250MG) and HCl 0.01 M (pH 2) for 48 h at 37 °C. Following incubation, the pH of the solution was adjusted to 7.2 with 1 M of NaOH 1:1 (Thermo Fisher Scientific, Waltham, MA, USA) and incubated at 45 °C for 30 min, vortexing the tube every 5 min. Finally, the pH was adjusted to 7.2 with ice-cold PBS.

The total protein concentration of the ECM solution was quantified using a standard colorimetric method based on the Lowry assay (DC™ Protein Assay Kit, Bio-Rad, Hercules, CA, USA) at a wavelength of 655 nm. Different quantities of ECM (40 µg, 80 µg, and 100 µg) were tested by performing the adhesion test using NSC cells (see Adhesion test paragraph below). A protein quantity of 80 µg was chosen and used for all subsequent experiments. A working ECM solution was prepared by diluting the stock ECM solution in PBS 1× to a final concentration of 2 mg/mL for the 2D coating. For the 3D ECM gel, the solution was first diluted in PBS 1× to a final concentration of 10 mg/mL. The gelation assay was performed on the ECM solution at 37 °C for 30 min in a standard cell culture incubator to find the lowest gelling concentration. Different dilutions were tested (1, 3, 6, 8 mg/mL).

### 4.3. DNA Extraction and Quantification

DNA extraction was performed to evaluate the efficacy of the decellularization process. DNA was extracted from pre- and post-decellularization SC tissues prior to the homogenization and lyophilization steps described above. From this step onwards, the procedures were carried out under a chemical hood. Pre- and post-decellularization SC samples were homogenized with a T 25 basic ULTRA-TURRAX^®^ homogenizer in 900 µL QIAzol Lysis Reagent (Qiagen, Hilden, Germany). Following homogenization, the samples were placed on ice for 30 s, 180 µL of chloroform was added, mixed for 3 min, and centrifuged at 12,000× *g* for 15 min at RT to obtain three phases. The apical aqueous phase and basal organic phase were discarded, and the intermediate phase containing nucleic acids collected, mixed with 2 volumes of 100% EtOH and 0.5 volume of ammonium acetate 7.5 M, and centrifuged at 12,000× *g* for 5 min at 4 °C. The pellet was resuspended in 70% EtOH and centrifuged at 12,000× *g* for 2 min at 4 °C. The supernatant was discarded, and the EtOH allowed to evaporate completely at RT. The pellet was resuspended with 500 mL of 8M NaOH to solubilize the DNA, and DNA quantification was performed at 260 nm (NanoDrop™ 2000, Thermo Fisher Scientific, Waltham, MA, USA).

### 4.4. Cell Cultures

Rat embryonic stem cells (RESC) are a cell line derived from 4.5 days post coitum blastocysts as previously described [26] and cultured in a monolayer (RESC single cells; RESC-sc). RESC-sc were cultured in RESC medium composed of DMEM-F12 with L-Glutamine (Merck, Darmstadt, Germany, cat no. D8437), 10% FBS ES quality (Gibco, Waltham, MA, USA, cat no. 16141079), 1% MEM/NEAA (Gibco, cat no. 11,140-050), 0.1 µM β-Mercaptoethanol (Gibco, cat no. 21985023), 1% penicillin/streptomycin (P/S; 100 U/mL^−1^/100 µg/mL^−1^, Gibco, cat no. 15070-063), and 0.1% Nucleoside mix (Merck, cat no. ES-008-D). Cells were plated at a seeding density of 5500 cells/well in 96-well plate for the adhesion test (20 min) and viability tests at 1 and 2DIV.

For the NSCs and NSC-derived OPC cultures, fetal NSCs from embryos at 13.5 days of gestation were isolated following previously published protocols [13]. Briefly, the embryo heads were placed in a Petri dish containing PBS 1× with 1% P/S. Under a dissection microscope, the brains were isolated from the skulls using a lancet and placed upright on the plate. The meninges were carefully detached using forceps, and the olfactory bulbs were removed. The forebrains were then collected in a 1.5 mL tube containing non-enzymatic dissociation buffer (Sigma-Aldrich, St. Louis, MO, USA) and incubated at 37 °C for 15 min. The tissues were then pipetted several times for mechanical dissociation and centrifuged at 400× *g* for 5 min at RT. The cellular pellet was resuspended in serum-free NSC medium composed of DMEM/F12 GlutaMAX 1× (Thermo Fisher Scientific, cat no.10565018), 8 mmol/L HEPES (Gibco, cat no. 15630056), 100 U/100 µg P/S (Gibco, cat no. 15070-063), 1× B27 (without retinoic acid; Gibco, cat no. 12587010), and 1× N − 2 (Gibco, cat no. 17502048). The following growth factors were added: 10 ng/mL bFGF (Gibco, cat no. PHG0024) and 10 ng/mL EGF (Gibco, cat no. PHG0311L). The resulting cells were plated at a seeding density of 10 cells/µL in a T25 flask (Corning, New York, NY, USA) kept in a vertical position to avoid cell adhesion to the flask surface. The half medium was changed and growth factors added every three days, and the primary neurospheres were allowed to proliferate until they reached a diameter of about 100 µm.

To obtain the secondary neurospheres and OPC-enriched spheres, primary neurospheres were centrifuged at 400× *g* for 5 min and resuspended in 1 mL of NSC medium, and the pellet was mechanically dissociated by pipetting. To obtain the secondary neurospheres, cells were counted and plated at a seeding density of 10 cells/µL in NSC medium with 10 ng/mL bFGF (Gibco, cat no. PHG0024) and 10 ng/mL EGF (Gibco, cat no. PHG0311L). To obtain the OPC-enriched spheres, cells were seeded at the same density but cultured in NSC medium with 10 ng/mL bFGF (Gibco, cat no. PHG0024) and 10 ng/mL PDGF (Gibco, cat no. PHG0035) (OPC medium).

Both types of spheres were mechanically dissociated with single cells when they reached a diameter of 100–200 µm. Then, 96-well plates were coated with poly-D, L-ornithine (50 µg/mL; Merck, cat no. P0421-100MG)/laminin (5 µg/mL; Merck, L2020) for control groups; with 80 µg/well ECM (normal, 2dpl, and 47dpl) for experimental groups. The 2D ECM coating solution was dispensed on culture plates and left open under a laminar flow hood overnight until complete evaporation. Following mechanical dissociation, cells from both types of spheres were counted and plated as follows: to derive OPCs from oligospheres, single cells were plated at a density of 3000 cells/cm^2^, while cells derived from secondary neurospheres were plated at a seeding density of 10,000 cells/cm^2^.

Cells derived from secondary neurospheres were cultured with NSC medium, while the following protocol was performed to obtain the oligodendrocyte differentiation from oligospheres (NSC-derived OPCs).

To induce oligodendrocyte differentiation and maturation, after 3 days of culture, the OPC medium was replaced with the oligodendrocyte differentiation medium, consisting of NSC medium with the addition of 50 nM T3 (Sigma-Adlrich; cat no. T2752), 10 ng/mL CNTF (Gibco, cat no. PHC7015), 1× N-acetyl-L-cysteine—NAC (Thermo Fisher Scientific, cat no. A15409.36). Half medium was changed every 2–3 DIV.

### 4.5. 3D ECM Gel Cell Culture

NSCs and NSC-derived OPCs were seeded on a 2D ECM coating for each experimental group as described above. After 24 h, the NSC medium was removed, and the ECM gel (8 mg/mL) matching the experimental group (ECM-Norm, ECM-2dpl, ECM-47dpl) was added on top of the ECM 2D-derived cultures and incubated for 30 min at 37 °C in a cell culture incubator for gelation. After incubation, the NSC medium was dispensed into each well, and the half medium was changed every 2-3 DIV until 12 DIV for NSC-derived OPCs, and 21 DIV for the NSC culture.

### 4.6. Adhesion Test

An adhesion test with NSCs was performed to establish the optimal quantity of ECM for use in all subsequent experiments. The test was carried out by seeding NSCs at a density of 10,000 cells/well in 96-well plates coated with different quantities of ECM-Norm (0 µg, 40 µg, 80 µg, and 100 µg) as described above. Cells for the control condition were seeded on poly-D, L-ornithine/laminin coating. Seeded plates were incubated in a cell culture incubator for 20 min, then fixed with cold 4% paraformaldehyde (PFA) at RT. At the end of incubation, two 10 min washes with 1× PBS were performed, and cells were stained with Hoechst 33258 nuclear dye (1 μg/mL; Invitrogen, cat no. H3569) in 0.3% Triton PBS 1× for 30 min at 37 °C. Wells were washed twice with PBS 1× (10 min per rinse) and kept in PBS 1× at +4 °C until analysis.

To test the different experimental ECM groups (ECM-Norm, ECM-2dpl and ECM-47dpl), an adhesion test was performed on RESC-sc by seeding 5500 cells/well in 96-well plates coated with 80 µg of each ECM and on gelatin coating (Merck, cat no. ES-006-B) as the standard culture control condition. RESC medium was used. After 20 min, cells were fixed as described above.

### 4.7. MTT Viability Assay

NSCs and NSC-derived OPCs were seeded in 96-well plates containing coatings from each experimental group (PO-Lam, ECM-Norm, ECM-2dpl, and ECM-47dpl) at a density of 10,000 and 3000 cells/well, respectively. After 3 and 7 DIV, cell viability was measured by MTT (Thiazolyl Blue Tetrazolium Bromide, Sigma-Aldrich) colorimetric assay, according to the manufacturer’s instructions. Briefly, the culture medium was removed, 100 µL of MTT solution (0.5 mg/mL) in OptiMEM (Gibco, cat no. 11058021) was added to each well, and cells were incubated in a cell culture incubator (37 °C, 5% CO_2_) for 3 h. Following incubation, 100 µL of solubilization solution (isopropanol 80%, HCl 1M 10%, Triton 10%) was added to each well, and the plates incubated for 1 h at RT under shaking. Absorbance was detected by a plate reader (Model 680, microplate reader, Bio-Rad, Hercules, CA, USA) at 570 nm.

### 4.8. LDH Activity Assay

Medium from NSCs and NSC-derived OPCs seeded at 20,000 cells/well on an 8-chamber slide from each experimental group (PO-Lam, ECM-Norm, ECM-2dpl, and ECM-47dpl) was collected at 1, 3, and 7 DIV, and LDH activity was measured by PIERCE LDH Cytotoxicity Assay Kit (Thermo Fisher Scientific, cat no. 88953), following the manufacturer’s instructions. Absorbance was detected by a plate reader (Bio-Rad Model 680, microplate reader) at 655 nm.

### 4.9. Immunocytochemistry

ICC was performed following blocking with a solution of Donkey Serum 1% and BSA 1% in PBS 1×, pH 7.4, for 1 h at RT. Following incubation, the 96-well plates and 8 chamber slides were incubated overnight at 4 °C with primary antibodies diluted in Triton 0.3% in PBS 0.01%, pH 7.4. The following primary antibodies were used: rabbit anti-Myelin Basic Protein (MBP) (1:250 Proteintech, cat no. 10458-1-AP) to mark mature oligodendrocytes; rabbit anti-Glial Fibrillary Acid Protein 174 (GFAP) (1:1000, Dako, Glostrup, Denmark, cat no. Z0334); mouse anti-CNPase (1:300, Millipore, cat no. MAB326R); mouse anti-βIII-tubulin (1:1000, Santa Cruz Biotecnhnology, Dallas, TX, USA, cat no. sc-51670); mouse anti-MAP2 (1:500, Dako, cat no. A32459), rabbit anti-Synaptophysin (SYN) (1:300, Dako), and rabbit anti-Oct4 (1:400, Abcam, Cambridge, UK, cat no. AB19857). The secondary antibodies used were donkey anti-rabbit IgG RRX (1:500, Jackson Immunoresearch, West Grove, PA, USA, cat no. 711-295-152), donkey anti-mouse AlexaFluor488 (1:500, Invitrogen Carlsbad, cat no. A21202), and donkey anti-rabbit AlexaFluor488 (1:500, Jackson Immunoresearch, cat no. 711-546-152). For nuclear staining, 1 μg/mL Hoechst 33258 (Invitrogen, Cat no. H3569) was added during incubation of the secondary antibodies.

For RESC-sc staining, Phalloidin-iFluor 647 (1:1000, Abcam; Code: AB176759) was also used. It was diluted in 1% BSA/PBS 1× and permeabilization performed with 0.3% Triton-X100/PBS for 10 min at RT before staining. Permeabilization and Phalloidin incubation were performed between the incubations with primary and secondary antibodies.

### 4.10. Microscopy and Image Analysis

The Cell Insight™ CX5 HCS platform (Thermo Fisher Scientific) was used to analyze the 96-well plates for the quantification of cell number, cell adhesion, cell death (condensed, pycnotic nuclei), and cell positivity to Oct4 (RESC-sc). Briefly, the software (HCS Studio v 6.6.0, Thermo Fisher Scientific) identifies each cell as an object by recognizing nuclear staining. It analyzes the nucleus size and Hoechst stain intensity to determine the percentage of small, high-intensity condensed nuclei in relation to the total number of nuclei in the well (% of pycnotic nuclei). The software also detects marker-specific staining in the cell body through the “Compartmental analysis” BioApplication algorithm to quantify the percentage of marker-positive cells (Oct4). The HCS system enables a comprehensive analysis of the entire culture, eliminating any operator bias associated with the selection of random representative fields. In each replicate, 10,000 to 20,000 cells per well were analyzed.

The histological images of the SC prior to and following decellularization were acquired using a Nikon Microphot-FXA light optical microscope fitted with a Nikon DXM1200F CCD camera (Nikon) with different magnifications (4×, 10×, 20×, 40×). A Nikon Eclipse E600 microscope (Nikon Instruments Europe BV, Amsterdam, Netherlands) with a QImaging Retiga 20002V digital CCD camera (QImaging, Surrey, BC, Canada) was used to capture representative Hoechst images.

Confocal microscopy was used to analyze the clusters on the 2D coating and the 3D ECM gel in 8-chamber slides. The slides were analyzed with an A1R confocal system (Nikon, Minato, Tokyo, Japan) using a diode laser system (405 nm λ output), air-cooled argon-ion laser (488 nm λ output), yellow diode-pumped laser (561 nm λ output), and a diode laser (638 nm λ output). Images were acquired using 20× and 40× lenses with 512 × 512 or 1024 × 1024 resolution, and all z-stacks were collected in compliance with optical section separation (z-Interval) values suggested by the NIS-Elements AR 3.2 software (1 μm).

### 4.11. Statistical Analysis

Data are reported as mean ± SEM. Prism software (v 9.0; GraphPad Software, Boston, MA, USA) was used for statistical analyses and graph generation. Data were tested for normality using the D’Agostino-Pearson test, showing normal distribution for all analyzed data. ANOVA (one- and two-way) and Dunnett’s multiple comparison post hoc test were performed to compare more than two groups, and results were considered significant when the probability of their occurrence as a result of chance alone was less than 5% (*p* < 0.05).

## Data Availability

The original contributions presented in this study are included in the article/Supplementary Material. Further inquiries can be directed to the corresponding author(s).

## Supplementary Materials

The following supporting information can be downloaded at: https://www.mdpi.com/article/10.3390/ijms26093969/s1.

## Abbreviations

The following abbreviations are used in this manuscript (alphabetical order):

βIII-tub	beta-III-tubulin
CNPase	cyclic-nucleotide 3′-phosphodiesterase
CNS	central nervous system
CSPG	chondroitin sulfate proteoglycans
DIV	days in vitro
Dpl	days post lesion
ECM	extracellular matrix
GAG	Glycosaminoglycans
GFAP	glial fibrillary acidic protein
HCS	high-content screening
LDH	lactate dehydrogenase
MAP2	microtubule-associated protein 2
MMP	matrix metalloproteinases
Norm	normal, no spinal cord injury
NSC	neural stem cells
OL	oligodendrocyte
OPC	oligodendrocyte precursor cell
PG	proteoglycans
RESC	rat embryonic stem cells
RESC-sc	Rat embryonic stem cells, monolayer
SC	spinal cord
SCI	spinal cord injury
SYN	synaptophysin
