# Supplementary material for: The Influence of Pathological Extracellular Matrix on the Biological Properties of Stem Cells: Possible Hints for Cell Transplantation Therapies in Spinal Cord Injury
Source: Int J Mol Sci. 2025 Apr 23;26(9):3969. doi: 10.3390/ijms26093969 (PMC12071833; doi:10.3390/ijms26093969)
Supplement: Supplementary file 1 [file ijms-26-03969-s001.zip › Supplementary Materials_CQ-VAB.pdf]

Supplementary Materials

Article

The influence of pathological extracellular matrix on the biological properties of stem cells: possible hints for cell transplantation therapies in spinal cord injury.

Giuseppe Alastra [1, †], Corinne Quadalti [2, †], Vito Antonio Baldassarro [1, 3], Alessandro Giuliani [1], Luciana Giardino [1, 2], Laura Calzà [2, 4, *]

[1] Department of Veterinary Medical Sciences (DIMEVET), University of Bologna, Ozzano dell'Emilia, Bologna, Italy

[2] Department of Pharmacy and Biotechnology (FABIT), University of Bologna, Bologna, Italy

[3] Interdepartmental Centre for Industrial Research in Health Sciences and Technology ICIR HST, University of Bologna, Bologna, Italy

[4] Montecatone Rehabilitation Institute, Montecatone (BO), Italy

* Correspondence: laura.calza@unibo.it

[†] These authors contributed equally to this work

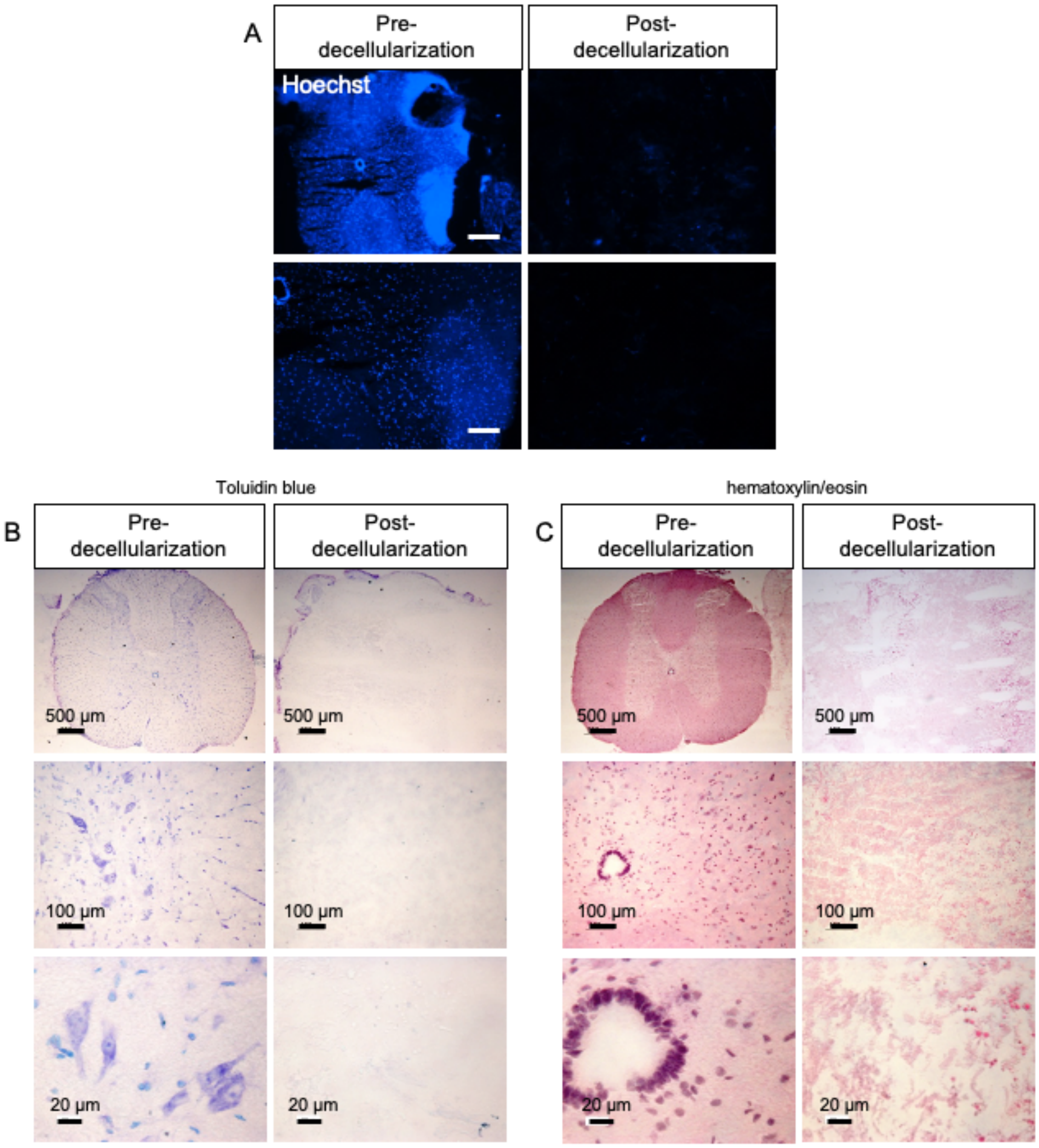

Supplementary Figure S1. Histological staining of spinal cord before and after decellularization.

(A) Representative micrographs of SC tissues labelled with the nuclear dye Hoechst 33258 pre- and post-decellularization, acquired via epifluorescence microscopy. Scale bar: 500 µm.

(B) Representative micrographs obtained via Toloudine blue and (C) hematoxylin/eosin staining of non-decellularized control tissue and decellularized one. The micrographs were obtained using a light optical microscope Nikon Microphot – FXA equipped with a CCD camera Nikon DXM1200F (Nikon).

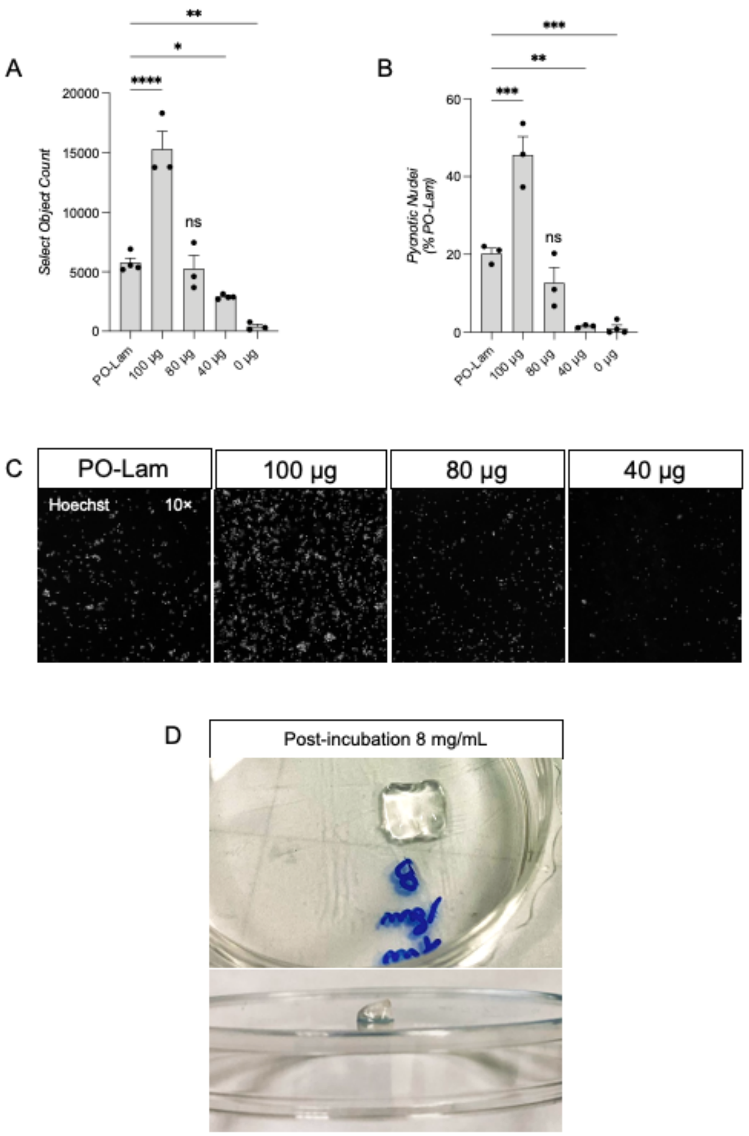

Supplementary Figure S2. Assessing the optimal ECM concentration.

(A – B) Graphs show the number of cells (A) and percentage of pycnotic nuclei (B) of NSCs after 20 min from seeding to evaluate the adhesion test.

(C) Representative images of HCS-derived NSCs adhesion test. Cells were seeded on PO-Lam or ECM 2D coating at 100, 80, 40, 0 µg and, after 20 min, fixed and stained with Hoechst 33258.

(D) Representative pictures of the 8 mg/mL gel after incubation at 37°C.

Statistical analysis: One-way ANOVA followed by Dunnett's post hoc test was performed for adhesion test graphs (A-B). One-way ANOVA, $F(4, 12)=249.99$, $p<0.0001$; Dunnett post-test, $p<0.0001$ for PO-Lam vs 100 µg, Dunnett post-test, $p=0.0452$ for PO-Lam vs 40 µg; Dunnett post-test, $p=0.0011$ for PO-Lam vs 0 µg. Asterisks represent differences between ECM-Norm (100%) and ECM-2dpl and ECM-47dpl coating (* $p < 0.05$; *** $p < 0.001$, **** $p < 0.0001$).

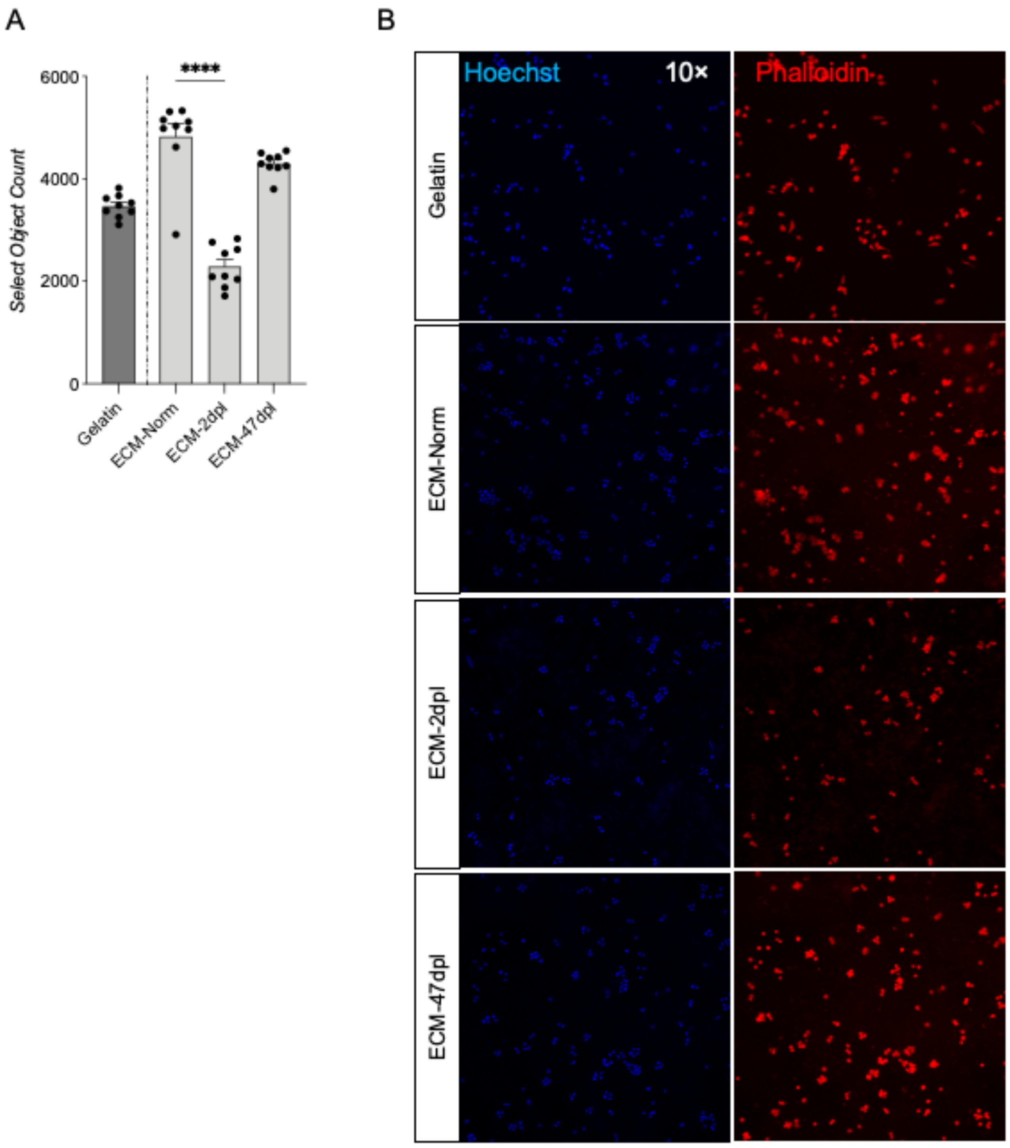

Supplementary Figure S3. Effect of 2D ECM-coating on RESC-sc adhesion.

(A) Graph shows the number of RESC-sc after 20 min from seeding to evaluate the adhesion test on gelatin, ECM-Norm, ECM-2dpl and ECM-47dpl.

(B) Representative HCS images of RESC-sc adhesion test stained with Hoechst and Phalloidin (10× magnification).

Statistical analysis: One-way ANOVA followed by Dunnett's post hoc test was performed for adhesion test graphs (A-B). One-way ANOVA, $F(4, 12)=249.99$, $p<0.0001$; Dunnett post-test, $p<0.0001$ for PO-Lam vs 100 µg, Dunnett post-test, $p=0.0452$ for PO-Lam vs 40 µg; Dunnett post-test, $p=0.0011$ for PO-Lam vs 0 µg. Asterisks represent differences between ECM-Norm (100%) and ECM-2dpl and ECM-47dpl coating (* $p < 0.05$; *** $p < 0.001$, **** $p < 0.0001$).

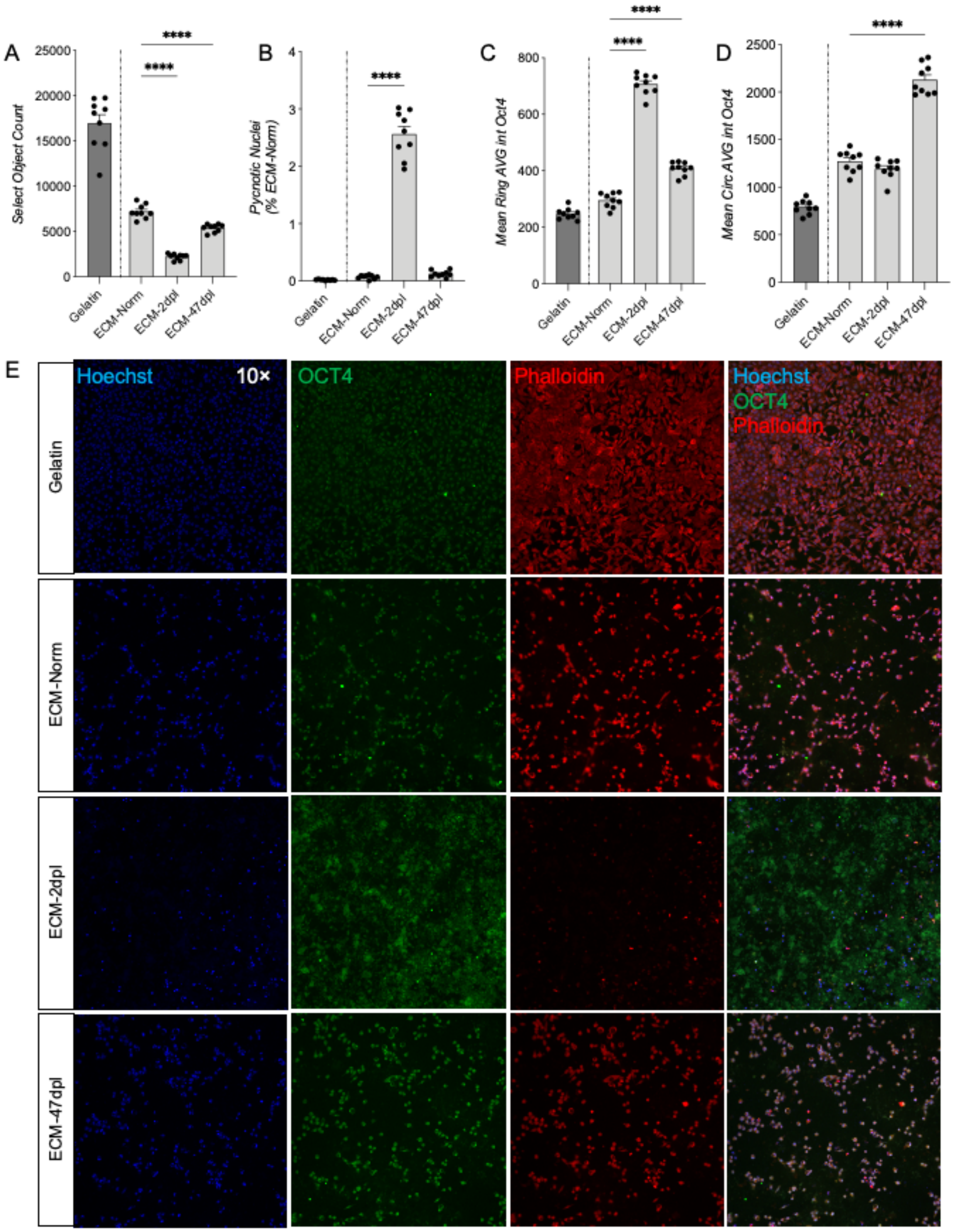

Supplementary Figure S4. Effect of 2D ECM-coating on RESC-sc at 2 DIV

(A – D) Graphs show the analysis of the number of cellsper well (Select Object Count, A), cell death (percentage of condensed nuclei, B), pluripotency as positivity to OCT4 marker in perinuclear (Ring AVG, C) and nuclear (Circ AVG, D) cell compartment of RESC-sc seeded on 2D coating (gelatin, ECM-Norm, ECM-2dpl and ECM-47dpl) after 2 DIV.

(E) Representative HCS images of RESC-sc cultures at 2 DIV, stained with Hoechst, Oct4 and Phalloidin seeded on gelatin, ECM-Norm, ECM-2dpl and ECM-47dpl.

Statistical analysis: for Select Object Count: One-way ANOVA $F(2, 24)=193.1$, $p<0.0001$; Dunnett post-test, $p<0.0001$ for ECM-Norm vs ECM-2dpl; Dunnett post-test, $p<0.0001$ for ECM-Norm vs ECM-47dpl. For Pycnotic Nuclei: One-way ANOVA $F(2, 24)=330.3$, $p<0.0001$; Dunnett post-test, $p<0.0001$ for ECM-Norm vs ECM-2dpl; for Ring AVG OCT4: One-way ANOVA $F(2, 24)=483.2$, $p<0.0001$; Dunnett post-test, $p<0.0001$ for ECM-Norm vs ECM-2dpl; Dunnett post-test, $p<0.0001$ for ECM-Norm vs ECM-47dpl; for Circ Ring AVG OCT4 One-way ANOVA $F(2, 24)=149.0$, $p<0.0001$; Dunnett post-test, $p<0.0001$ for ECM-Norm vs ECM-47dpl.

Asterisks represent differences between ECM-Norm (100%) and ECM-2dpl and ECM-47dpl coating (**** $p < 0.0001$).
